# Rasch analysis for development and reduction of Symptom Questionnaire for Visual Dysfunctions (SQVD)

**DOI:** 10.1038/s41598-021-94166-9

**Published:** 2021-07-21

**Authors:** Mario Cantó-Cerdán, Pilar Cacho-Martínez, Francisco Lara-Lacárcel, Ángel García-Muñoz

**Affiliations:** 1 Departamento de Óptica, Farmacología y Anatomía, Universidad de Alicante, Alicante, Spain; 2 Departamento de Oftalmología, Optometría, Otorrinolaringología y Anatomía Patológica, Universidad de Murcia, Murcia, Spain

**Keywords:** Refractive errors, Vision disorders, Eye manifestations

## Abstract

To develop the Symptom Questionnaire for Visual Dysfunctions (SQVD) and to perform a psychometric analysis using Rasch method to obtain an instrument which allows to detect the presence and frequency of visual symptoms related to any visual dysfunction. A pilot version of 33 items was carried out on a sample of 125 patients from an optometric clinic. Rasch model (using Andrich Rating Scale Model) was applied to investigate the category probability curves and Andrich thresholds, infit and outfit mean square, local dependency using Yen’s Q3 statistic, Differential item functioning (DIF) for gender and presbyopia, person and item reliability, unidimensionality, targeting and ordinal to interval conversion table. Category probability curves suggested to collapse a response category. Rasch analysis reduced the questionnaire from 33 to 14 items. The final SQVD showed that 14 items fit to the model without local dependency and no significant DIF for gender and presbyopia. Person reliability was satisfactory (0.81). The first contrast of the residual was 1.908 eigenvalue, showing unidimensionality and targeting was − 1.59 logits. In general, the SQVD is a well-structured tool which shows that data adequately fit the Rasch model, with adequate psychometric properties, making it a reliable and valid instrument to measure visual symptoms.

## Introduction

The presence of any type of visual dysfunction, refractive, accommodative and/or binocular, may cause the appearance of visual symptoms that affect the level of comfort of a patient when performing a visual task^1^.

The scientific literature shows disparity regarding the symptoms associated with the diagnosis of these visual anomalies, the way for asking the patient about them and how to classify the degree of severity^2,3^. In a scoping review published by García-Muñoz et al.^3^ it was shown that there are differences when compiling information and classifying symptoms of the anomalies. In that review it was shown that the most common symptoms related to these anomalies may include diplopia, movement or blinking of the words in near vision, headaches, visual fatigue and blurred vision and that these symptoms could be classified into 34 different categories, all of them fundamentally related to near vision. In this review it was also observed that there are a total of 11 questionnaires in the scientific literature^4–15^. Some use dichotomous questions and others multiple choice with up to 5 responses per item. Of the 11 questionnaires, only three of them were validated^5,10–12^. One of them (the Conlon survey) referring to visual discomfort not associated to any specific dysfunction^5^ and the CISS V-15 and its version for parents (CIRS parent version) to convergence insufficiency^10–12^.

Scientific literature has also shown specific validated questionnaires to collect the different symptoms which may be associated to computer screens or video terminals^16,17^, although this type of questionnaires does not cover all situations of everyday life since they are specific to these devices.

As it can be seen, no symptom questionnaire is currently available asking questions which could be related to any type of visual dysfunction (refractive, accommodative or binocular) which may provoke symptoms. The available questionnaires have been developed for specific visual anomalies such as convergence insufficiency^11^, have been related to visual discomfort in general^5^ or have been focused on particular samples as computer users^16,17^ and only some of them have been psychometric validated using Rasch analysis^5,16,17^. However, we consider it should be interesting to have a questionnaire which would allow the detection of any visual symptoms associated with any type of visual dysfunction. The instrument would broadly cover all cause of these symptoms, including refractive, accommodative and binocular. That is, a questionnaire which could detect the presence or absence of visual symptoms and could measure their frequency. This tool would be useful for clinicals as they could accurately detect the presence of symptoms, supporting the diagnosing process. Furthermore, this questionnaire could be used by professionals to calibrate the visual symptomatology when monitoring the treatment of visual anomalies. Clinicians would be able to detect the improvement of patients by means of symptoms’ disappearance or through the decrease of their frequency.

In this sense, we are developing a questionnaire that covers this need. At the beginning of the research, we developed a pilot questionnaire using Delphi methodology in which various experts discussed the inclusion of different categories of visual symptoms in a questionnaire^18^. This pilot questionnaire on visual symptomatology is the starting point for this research and for its development, a psychometrical evaluation using Rasch analysis is needed. There are several statistical methods to psychometrically analyze an instrument: The Classical Test Theory (CTT) or the Item Response Theory (IRT). CTT has several limitations which have given place to use the IRT. IRT refers to probabilistic measurement models according to the number of parameters analyzed so that Rasch analysis is used when 1-parameter is studied, that is, when unique construct exists. The strength of Rasch methodology is that it allows conjoint measurement of persons and items on the same dimension or construct^19^. This method is recommended in this type of instruments^20^, as it provides an idea of the internal consistency of the scale and can match the item difficulty with the person's ability^21^.

Therefore, the aim of this study is to develop the Symptom Questionnaire for Visual Dysfunctions (SQVD) and to perform a psychometric analysis using Rasch method to obtain an instrument which allows to detect the presence and frequency of visual symptoms related to any visual dysfunction.

## Methods

### Design of the pilot questionnaire

In this study the Symptom Questionnaire for Visual Dysfunctions (SQVD) was developed. We started with a pilot version of 33 items on which a psychometric reduction of items was made to obtain the final questionnaire.

The study followed the tenets of the Declaration of Helsinki, and informed consent was obtained from all subjects after explanation of the nature of the study. If patient was under 18 years old, informed consent was obtained from the parent and/or legal guardian. This research was approved by the ethical committee of the University of Alicante.

The development of the SQVD was based on a 33-item pilot questionnaire which was developed according to scientific evidence. García-Muñoz et al.^3^ reported the symptoms described by patients in the scientific literature (by means of clinical history or questionnaires) when considering any type of visual anomalies and grouped them into 34 categories of symptoms. Using a Delphi methodology, it was discussed the inclusion of these 34 different categories of visual symptoms in a questionnaire of visual symptomatology, for which experts considered to add other symptom categories^18^ so that these results were the origin of this pilot questionnaire.

Thus, considering the Delphi results, and to ensure the validity for the content development aspect and seek patient input in that content development phase, we created a cognitive pre-test^22^ which had 47 questions of multiple choice answers on a Likert scale of 4 answers (No, Occasionally, Often and Almost always), which measured the frequency of each item. This preliminary instrument with 47 questions was evaluated in a clinical sample of 30 patients with ages between 18 and 71 years old (mean age 29.83 ± 11.07 years old) from an optometric clinic.

In addition to the 47 items of the instrument, a semi-structured interview was conducted, where the patients were asked about those 47 questions that the initial questionnaire had. In this way, they were asked if the questionnaire was too long, if the way to answer the questions was well understood, if the mode of representing the frequency of the response was well understood, or if there were any questions that they had not understood (specifying which one). In addition, they were asked about those questions that we initially thought they could say the same, to see what patients felt. We also asked if they believed it would be better understood with other words to ask about frequency. And also, at the end, patients were asked if they wanted to give their opinion or any suggestion about the items.

Once obtained all of this information, a first qualitative reduction of items was done, eliminating repeated questions and making the changes suggested by the patients, so that we obtained the 33-item pilot scale. These 33 items were randomly distributed into the pilot instrument. They were written as multiple-choice questions on a Likert scale of 4 responses which measured the frequency of the item. The meaning of each answer (which was described in the instructions of the questionnaire) was the following:

- No: the symptom never occurs.
- Occasionally: the symptom occurs at least once every 15 days.
- Often: the symptom occurs once or twice a week.
- Almost always: the symptom occurs almost every day.

This pilot instrument was carried out on a consecutive population sample of 125 patients of an optometric clinic, aged between 15 and 84 (41.72 ± 14.92 years old). Regarding the inclusion criteria, subjects without any type of ocular pathology, dry eye were included. Of the clinical sample, 74 people (59.2%) were women and 51 (40.8%) men. 68 patients (54.4%) were non-presbyopic and 57 (45.6%) were presbyopic. According to visual anomalies, 95 patients had refractive dysfunctions, 18 binocular dysfunctions, 7 accommodative anomalies and 5 subjects did not have any visual anomaly.

### Rasch analysis

Rasch analysis is a probabilistic model that assumes items vary in difficulty^23^. It estimates the difficulty of items (*item difficulty*) and the relative abilities of the persons (*person ability*) associating them in a common invariant interval-level scale so that this allows an easy comparison of measures^24^. It transforms the ordered qualitative observations into quantitative interval level measures on a linear logit scale^25^. Item difficulty refers to the difficulty level of each item relative to other items in the scale^26^, so negative values indicate less severity and positive values show greater severity. Person ability refers to the ability level of each person relative to other persons in the sample^26^.

Rasch analysis was carried out using Winsteps software (version 4.8.1). Rasch parameters analyzed included: functionality of the response categories, fit statistics, local dependency, differential item functioning (DIF), person and item reliability, unidimensionality, targeting and transformation table^27^.

As items were polytomous, a choice was needed between the Partial Credit Model (PCM) or the Andrich Rating Scale Model (ARSM) for Rasch analysis. PCM considers a different rating scale for each item, while ARSM assumes equal category thresholds across items. We applied the ARSM for polytomous responses after testing it against the PCM using the likelihood ratio statistic. As the test was not significant (*p* > 0.05), the ARSM was appropriate as all items shared the same rating scale^28^.

#### Functionality of the response categories

Rasch analysis also provides information about the best number of response categories in the scale. In order to analyze whether the category calibration increases in an orderly manner, response options were assessed with category probability curves (CPC)^29^. They show the likelihood that a subject with a specific person measure relative to item difficulty will select the category^20^. The threshold is the midpoint between adjacent response categories so that it reveals the point where the likelihood of choosing either response category is the same^30^. If a disordered threshold occurs, the situation must be amended collapsing the category needed into an adjacent category^20,31,32^. For detecting this situation, the Andrich threshold measure must be examined so that thresholds should be spaced at least 1.4 logits^29^.

#### Fit statistics

Rasch fit statistics, infit and outfit mean square are obtained to explore the compatibility of the data with the model^23,33^. They compare the predicted responses to those observed. *Infit* refers to information-weighted fit. It is more sensitive to the pattern inlying observations and less sensitive to outliers. *Outfit* indicates outlier-sensitive fit. It is more sensitive to outliers (atypical cases) so that allows to detect unusual events that occur unexpectedly. Infit and outfit mean squares (MNSQ) closer to 1 indicate a good fit to the model. They must have values between 0.70 and 1.30 logit range^20^. Values less than 0.70 indicate possible redundancy of items and values greater than 1.30 suggest that items may be measuring something different to the overall scale.

Accordingly, infit and outfit mean square were used to develop the item reduction of the initial instrument and when the reduction was done, the overall infit and outfit mean square of the final scale were obtained to test if the data fit the Rasch model for knowing if the model fit adequate, poor or excellent.

Item reduction criteria were applied using the guidelines described by several authors^20,23,30,34^. According to these criteria, items were eliminated using the following order of priority:

1. Items with values infit mean square outside 0.70 to 1.30.
2. Items with outfit mean square outside 0.70 and 1.30.
3. Items with a high proportion of missing data (> 50%).
4. Items with ceiling effect: a high proportion of responses in item end-response category (> 50%).
5. Items with a considerably different standard deviation of scores to other items.
6. Items with coefficients of skewness and kurtosis outside the range + 2 to − 2.

Item reduction was done by means an iterative process so that one item was removed at a time^23^. Thus, when an item was removed, fit to the model was reestimated consequently as it has been shown that fit is relative so that removal of items leads to variations in fit. Then, the item with the highest number of candidate criteria, ordered by priority was removed first.

#### Local dependency

Local dependency determines whether the response to any item has a direct impact on the response to any other item^35^. Local independence is a requirement of the model so that the dependence of items is analyzed to detect its violation with the Yen’s Q3 statistic. Hence, local dependency was examined using the residual correlation matrix. As it is stated by Christensen et al.^35^, no single critical value can be considered to indicate dependency. Simulations have shown that the Q3 critical value appears to be reasonably stable around a value of 0.2 above the average correlation. Therefore, it has been shown that any residual correlation > 0.2 above the average correlation would appear to indicate local dependency.

Accordingly, once performed the reduction of items, it was analyzed the local dependency to determine if there were redundant items which had also to be eliminated. If a pair of items had local dependency (when residual correlations exceeding the mean of all residual correlation by 0.20), items were removed or retained so that those items with an outfit MNSQ closed to 1 must be retained^36^.

#### Differential item functioning

Differential item functioning (DIF) is an analysis used to determine if an item measures a latent construct in the same way for different groups^24^. It represents the differences in item difficulty between respondent groups and it is important to assess it as DIF can affect fit to the model and may damage measures^27^. In this study DIF analysis was evaluated for gender and presbyopia (presbyopes and non-presbyopes subjects, considering presbyopia when the patient needed addition). Several authors have stated that mean differences in person measures between compared groups should be less than 1.0 logit^25^ so that when the value is greater than 1.0 logits, a notable DIF must be considered^37^. Accordingly, a statistically significant (*p* < 0.05) DIF contrast with a difference of more than 1.0 logit was considered for having DIF^24^. The Rasch-Welch t test method was used to determine the significance of the DIF contrast.

#### Person and item reliability

Rasch analysis also provides reliability (separation index) for both, person and item, showing the overall performance of the instrument^20^.

Reliability (separation index) means reproducibility of relative measure location, so that person and item reliability determine the replicability of the person and item locations along the trait continuum^24^. Accordingly, high reliability (of persons or items) means that there is a high probability that persons (or items) rated with high measures actually do have higher measures than persons (or items) estimated with low measure.

Person separation index is used to classify people, so that a low person separation implies that the instrument may not be enough sensitive to distinguish between high and low performs, showing that more items are needed. Similarly, item separation index is used to verify the item hierarchy, so that a low item separation implies that the sample is not large enough to prove the item difficulty hierarchy of the scale.

Thus, it has been suggested that person and item reliability can range between 0 and 1, which high values indicating better reliability. For person reliability a value of more than 0.80 is acceptable (person separation index > 2 logits) which it means that the measure can stratify the population into at least three groups based on their latent trait measure^38^. For item reliability a value of more than 0.90 (item separation index > 3 logits) is considered acceptable^20,39^.

#### Unidimensionality

Unidimensionality refers to the assumption that the items summed all together form a unidimensional scale^27^. It was explored by means of the principal component analysis (PCA) of the residuals, considering residuals the differences between observed data and the estimation of the model. The magnitude of the first contrast of the residual is an important indicator, so that this result should not be above 2 eigenvalue^27^.

#### Targeting

Rasch analysis also examines the targeting by means of the person-item map^40^. Targeting is the difference between the person ability mean and the item difficulty mean. The closer the person ability mean is to the item difficulty mean, the better the targeting. A difference of zero between both values will indicate a perfect targeting of the scale and a difference of more than 1 logit indicates mistargeting^30^.

#### Transformation table

Once a best fitting model has been found, Rasch analysis also allows to transform the ordinal scores of the questionnaire to an interval scale. This conversion table was obtained considering ordinal scores range of the scale. Then, the corresponding interval-level scores in logits and ordinal scale range were obtained.

## Results

**Figure 1:**
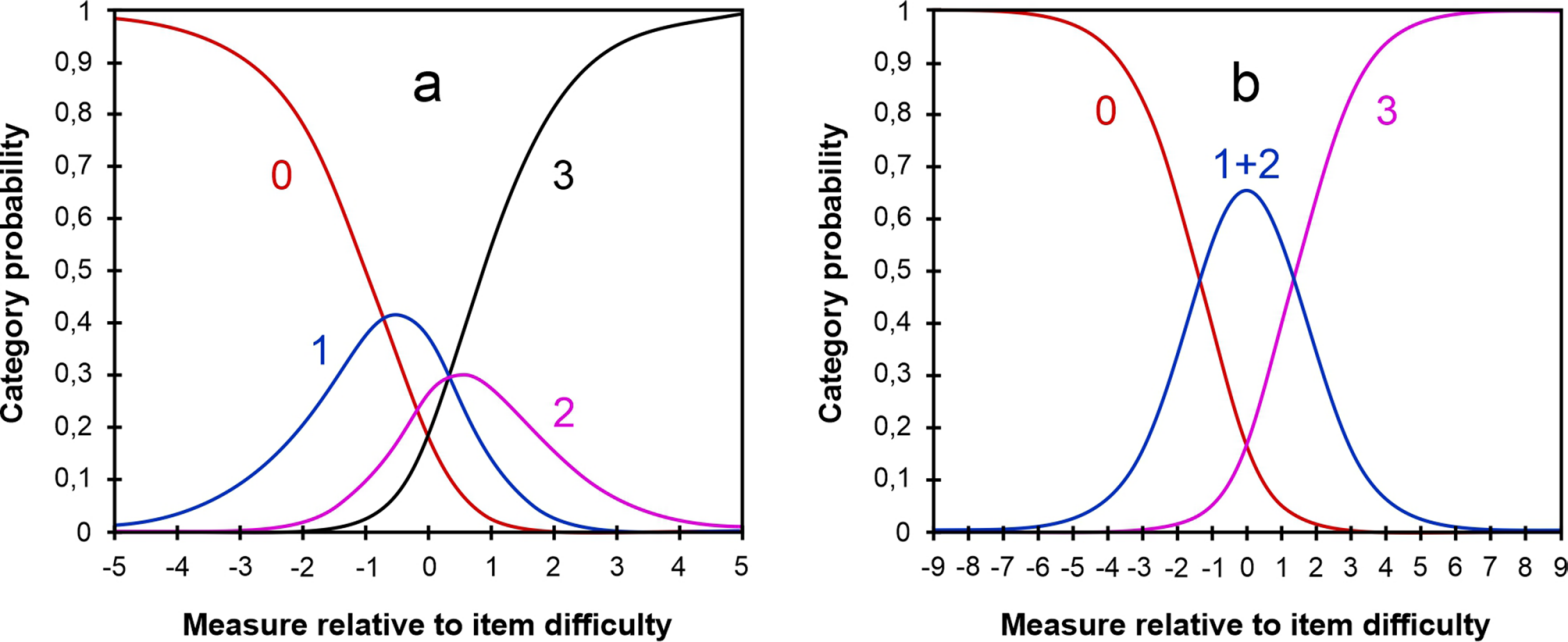
(**a**) Shows the category probability curves (CPC) for the instrument with 33 items and four response categories. Each curve in the CPC graph represents one response category (No = 0; Occasionally = 1; Often = 2; Almost always = 3). The point where two adjacent curves overlap is the threshold. At this intersection, it is the same likelihood of choosing one category or another. (**b**) CPC for the instrument with 33 items collapsing the categories 1 (Occasionally) and 2 (Often).

Figure 1a shows the CPC for the 33-item pilot SQVD with its four initial response categories. The threshold shown by the response categories 1 and 2 and the threshold of categories 2 and 3 corresponds to the same value of the person measure relative to item difficulty. This implies that the category calibration does not increase adequately, the thresholds are disordered, so that this situation had to be amended. For that, the Andrich thresholds were examined (Table 1). It shows that for the four initial categories, these values were not within the value of 1.4 logits between thresholds indicating that this premise is verified when categories 1 and 2 were collapsed. Accordingly, initial categories 1 (Occasionally) and 2 (Often) were collapsed into the category *Occasionally / Often*, resulting three category response options: 0 (No), 1 (Occasionally / Often) and 2 (Almost always). Figure 1b shows the CPC for the 33-item pilot version of SQVD with these three response categories.

**Table 1:** Andrich thresholds values (logits) for the 33-item pilot version of SQVD with its original four categories and collapsing categories.

	Category	Andrich thresholds
A	0	None
	1	− 0.67
	2	0.25
	3	0.42
B	0	None
	1 + 2	− 1.34
	3	1.34
C	0	None
	1	− 0.37
	2 + 3	0.37

A: results for 33-item pilot version with the initial four categories of response (0–1–2–3). B: results when collapsing the categories 1 and 2. C: results when collapsing categories 2 and 3.

Once the instrument had collapsed into three categories, the item reduction was analyzed. Using the criteria explained in the section of methods for reducing the instrument, 17 items were removed, and were the following: 3, 4, 5, 7, 9, 10, 11, 13, 16, 18, 20, 21, 22, 24, 27, 29, 30. Once removed these items, a version of 16 items was obtained.

With this version of the scale, the local dependency was performed to test if more items had to be removed. As the average correlation was 0.12, those residual correlation values > 0.32 (0.12 + 0.2) indicated dependency. Table 2 shows the residual correlation values greater than the value of 0.32 for the pair of items indicating dependency. It also indicates those items which were removed and retained using the local dependency criteria to reduce items. Once applying these criteria, items 28 and 31 had to be removed, so that the final questionnaire was comprised with 14 items. With these 14 items the local dependency was again analyzed, and no local dependency was obtained.

**Table 2:** Local dependency analysis by means of Yen’s Q3 statistic.

Residual correlation	Item	OUTFIT MNSQ	Result	Item	OUTFIT MNSQ	Result
0.41	31	0.88	Removed	33	1.01	Retained
0.37	28	0.92	Removed	33	1.01	Retained
0.34	25	0.95	Retained	31	0.88	Removed
0.33	28	0.92	Removed	31	0.88	Removed

Pair of items with a residual correlation > 0.2 above the average correlation (0.12) indicate dependency. In this case, residual correlation greater than 0.32 between them were shown. Items with an outfit MNSQ closed to 1 were retained. (MNSQ: mean square).

Table 3 shows the infit and outfit mean squares for the 14 items of the final instrument. It also shows DIF contrast results for the variables examined, for which there were no significant statistically differences (*p* > 0.05) regarding gender for all items (< 1 logits). With respect to the analysis of presbyopia, there were no significant statistically differences for all items (< 1 logits), except for the item 33.

**Table 3:** Item Rasch analysis results of the SQVD.

Item	Infit MNSQ	Outfit MNSQ	Gender DIF contrast	Presbyopia DIF contrast
1	0.99	0.96	0.37	0.33
2	1.04	0.97	0.32	0.13
6	0.98	0.91	0.29	0.49
8	0.98	0.95	0.00	0.08
12	1.01	1.00	0.53	0.10
14	0.96	1.06	0.33	0.89
15	1.03	1.06	0.37	0.71
17	0.78	0.74	0.37	0.00
19	1.19	1.24	0.19	0.53
23	0.86	0.85	0.19	0.05
25	1.10	0.99	0.36	0.21
26	0.98	1.07	0.24	0.13
32	0.84	0.95	0.13	0.00
33	1.18	1.13	0.07	1.71*

(MNSQ: Mean square statistics; DIF: Differential item functioning). *p < 0.05.

The overall infit and outfit mean square of the final scale are shown in Table 4, in which it can also be seen the person and item reliability values.

**Table 4:** Summary of the global fit statistics for person ability and item difficulty parameters for the SQVD.

Persons		Items		Reliability (Separation index)	
Infit MNSQ	Outfit MNSQ	Infit MNSQ	Outfit MNSQ	Person reliability (Separation)	Item reliability (Separation)
0.99	0.99	0.99	0.99	0.81 (2.11)	0.80 (2.06)

(MNSQ: Mean square statistics).

When exploring principal component analysis (PCA) of the residuals, the magnitude of the first contrast of the residual was 1.908 eigenvalue, showing the unidimensionality of the instrument.

**Figure 2:**
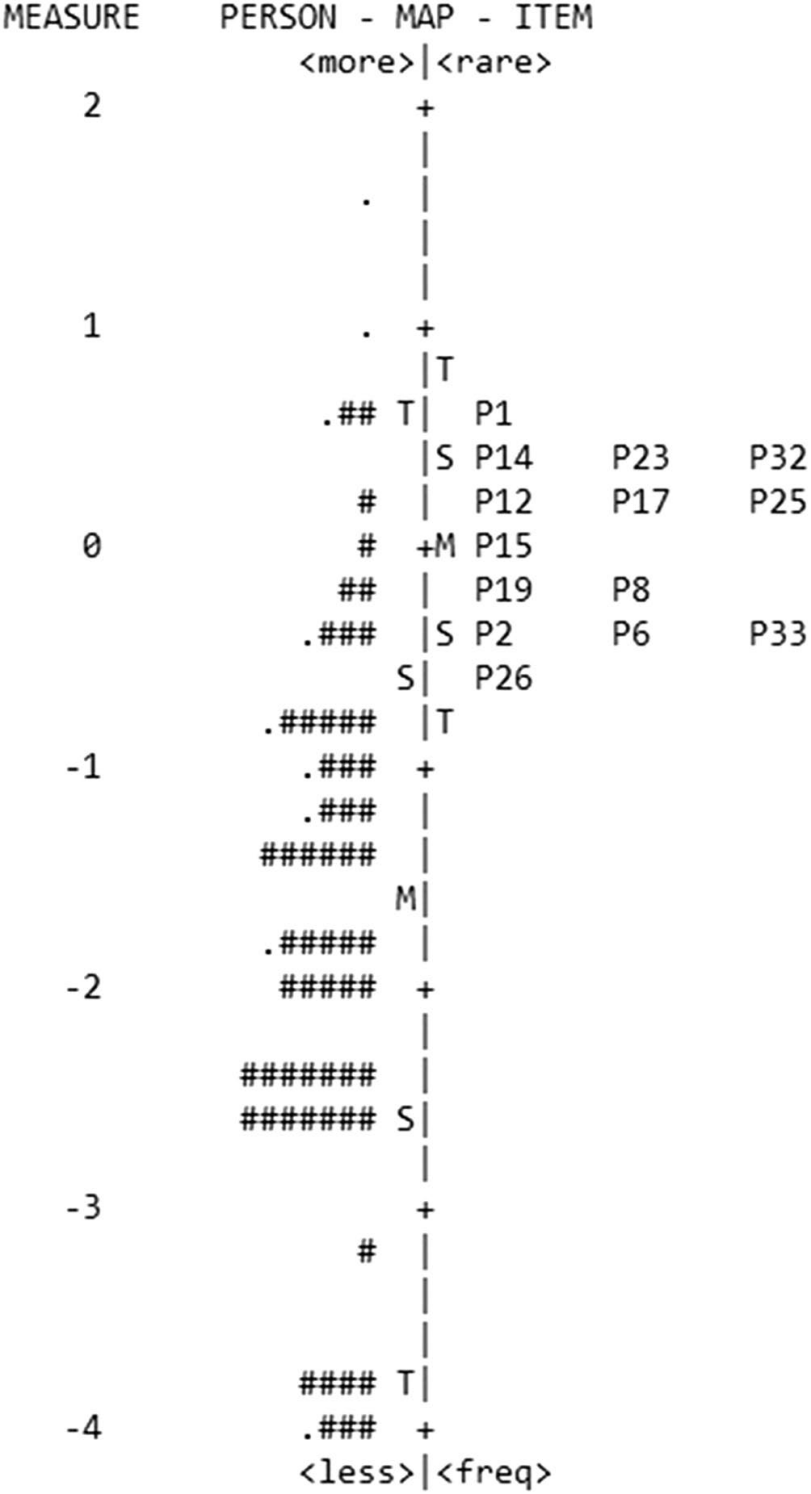
Person-item map for the SQVD. Patients are represented on the left of the dashed line by the symbol "#" (which represents 2 subjects) and "." (which indicates 1 subject). On the right of dashed line are illustrated the items of SQVD 14-item version with their number (Pnumber of item). M indicates the mean measure (on the left the person ability and on the right the item difficulty). S shows one standard deviation from the mean and T denotes two standard deviations. Higher ability for persons (higher frequency of symptoms) and more difficult items are on the top of the figure.

According to the targeting, the person-item map is shown in Fig. 2. Left-hand column indicates person ability (in logits), where those patients with higher ability are shown at the top of the figure. The mean person ability (showed in the figure as the left M) has a value of − 1.59 logits. The right-hand column indicates the item difficulty, for which the mean (right M) is always 0. Accordingly, more difficult items are on the top of the figure. With these results, the targeting of the scale was − 1.59 logits.

Transformation table results are shown in Table 5. It shows the ordinal scale and the corresponding interval-level scores. The ordinal scores range from 0 to 28 and corresponding interval scores in logits are included. Interval scores in logit units were rescaled into the ordinal scale range (0–28).

**Table 5:** Converting from a raw SQVD score (0–28) to an interval scale in logit units and using the original scale metrics.

Ordinal measure	Interval measure	
Raw score	Logit	Scale
0	− 5.19	0.00
1	− 3.93	3.39
2	− 3.17	5.45
3	− 2.69	6.75
4	− 2.32	7.74
5	− 2.01	8.57
6	− 1.74	9.30
7	− 1.50	9.96
8	− 1.27	10.59
9	− 1.04	11.18
10	− 0.83	11.76
11	− 0.62	12.33
12	− 0.41	12.89
13	− 0.21	13.45
14	0.00	14.00
15	0.21	14.55
16	0.41	15.11
17	0.62	15.67
18	0.83	16.24
19	1.04	16.82
20	1.27	17.41
21	1.50	18.04
22	1.74	18.71
23	2.01	19.43
24	2.32	20.26
25	2.69	21.25
26	3.17	22.55
27	3.93	24.61
28	5.19	28.00

## Discussion

Results of this study show that the SQVD has acceptable psychometric properties in a clinical population. Based on a previous research using Delphi methodology, the 33-item pilot instrument was reduced by Rasch analysis until the final version of 14 items was achieved. This final version of SQVD showed adequate fit statistics to the Rasch model, without local dependency or significant statistically DIF for gender and presbyopia, and an adequate reliability and unidimensionality.

The 33-item pilot version showed an inadequate order in the response categories, so it was necessary to collapse category 1 (occasionally) and 2 (often). Therefore, the SQVD response categories were reduced to three: no / occasionally-often / almost always, which allowed ordered thresholds with separations greater than 1.4 logits between them. This situation of collapsing categories has also been used by several questionnaires related to vision^16,17,25^.

According to the reduction of the scale, 19 items from the 33-item pilot version of SQVD were removed. These 19 items were related to reading problems, visual fatigue, diplopia, ocular problems, blurred vision and postural problems. Accordingly, the final version SQVD had 14 items, a number of items similar to those questionnaires showed in the scientific literature related to visual symptoms^5,10–12^. The 14 items that were finally included in the SQVD were related to blurred vision, binocular vision problems, ocular irritation, concentration difficulties, reading problems and headache.

All these 14 individual items demonstrated a satisfactory fit to the Rasch model, which could be considered productive for measurement (infit MNSQ between 0.78 and 1.19; outfit MNSQ between 0.74 and 1.24). Mean infit and outfit MNSQ for both subjects (mean infit 0.99 and outfit 0.99 logits) and items (mean infit 0.99 and outfit 0.99 logits) are within the range suggested by scientific literature^20,23,29,34,41^, so it can be considered that there is an adequate fit to the model. Furthermore, differential item functioning analysis showed that the SQVD had not significant statistically DIF by gender and presbyopia. There was only one item, item 33, which showed significant DIF for presbyopia. As this item is related to blurred near vision, it could argue that this symptom is more specific of a presbyopic population, so we consider that this item should not be removed from the questionnaire. In any case, DIF results allow to assume that the model and the set of item parameters are similar for all comparable groups.

According to the reliability, the person reliability result of 0.81 (person separation 2.11) was satisfactory, which implies the good internal consistency for the SQVD in this sample. The instrument could stratify the population into at least 3 groups based on the latent trait measured. However, the low item reliability of 0.80 (item separation 2.06) shows that it would be necessary a greater sample to prove the item difficulty hierarchy of the scale.

The basic assumption of the Rasch model^27^ was demonstrated for the SQVD as the principal component analysis (PCA) results of 1.908 eigenvalue proved the unidimensionality of the scale.

Targeting result of − 1.59 logits shows that the SQVD 14 item version has poor targeting, revealing a floor effect, situation clearly exhibited in Fig. 2 where it can be seen the important number of patients who are situated on the bottom of the figure. This might be explained by overall lower symptoms in the current sample, showing that in this sample, there is a greater number of subjects with less symptomatology. Other authors have shown that this situation is common in questionnaires related to symptoms, as many patients may not have symptoms^17,21^ or due to there is a tendency for subjects to underreport their discomfort^16^. These conditions are applicable to the SQVD, so accordingly, the targeting may be considered reasonable. In any case, it would be desirable for future studies to analyze samples that have higher levels of symptoms.

In this study it has been shown the conversion from ordinal to interval-level data^27^ without modifying the original response of the instrument. Using the conversion table provided here, users are able to increase the precision of the SQVD and thus confidence in any reported finding. It may be useful for clinical purposes, for example when consider reporting changes of the measure variable, as equal interval scaling allows to detect any variations. In addition to that, the conversion to interval scores facilitates the use of parametric statistics. However, it only may be used when there are no missing data, that is, when the completed data for all items are available for the assessed person.

The SQVD is difficult to compare with other visual symptoms questionnaires due to the differences in their purposes. Thus, the Conlon Visual Discomfort Scale Survey questionnaire^5^ is an instrument to measure the symptoms of visual discomfort, but this is different to visual symptoms related to the visual anomalies. Visual discomfort may be due to any cause, including for example the glare produced by a light source, so it is not comparable to the SQVD. Similarly, the CISS^10–12^ is a specific questionnaire only for a binocular anomaly (convergence insufficiency), so it cannot be applied to the others visual anomalies and therefore its categories of symptoms cannot be compared to those of the SQVD. Furthermore, the CISS instrument has not been validated using the Rasch model.

This study had some limitations. Although the pilot version of 33 items was distributed to 125 subjects, the sample size should be considered small. Several authors^42^ have suggested that the number of subjects could vary from 4 to 10 subjects per item, with a minimum of 100 subjects. However other authors^43^ have shown that there are not absolute rules for the sample size needed to validate a questionnaire.

However, the study has several strengths. One of them is related to the population characteristics of the sample. From a clinical point of view, an instrument as the SQVD has practical benefits for clinical purposes as it may be an aid to calibrate the patients’ symptoms who attend in an optometric clinic. Because the clinical population should be the target population of this instrument, it is a strength that its development had been done using a clinical sample. Furthermore, the starting point of the study is also another strength to consider. Thus, the meticulous process developed to determine which symptoms should be included in a questionnaire of visual symptoms (the previous Delphi method used)^18^, the detailed procedure developed to design the initial scale, including a previous questionnaire in other clinical sample and the comprehensive patient consultations, contributed to the strength of the study.

In conclusion, the SQVD is an instrument with 14 items which shows that data adequately fit the Rasch model, without local dependency or significant statistically DIF for gender and presbyopia, and an adequate reliability and unidimensionality. This allows to this instrument to have practical benefits not only for clinicians but researchers as it could be used to monitor symptoms in subject who are attended in a clinical center with any type of visual anomalies and supervise their treatment. Future studies should be done to confirm the SQVD behavior in a larger sample with patients more symptomatic.
